# Detection of phosphatidylserine-positive exosomes as a diagnostic marker for ovarian malignancies: a proof of concept study

**DOI:** 10.18632/oncotarget.14795

**Published:** 2017-01-22

**Authors:** Jayanthi Lea, Raghava Sharma, Fan Yang, Hong Zhu, E. Sally Ward, Alan J Schroit

**Affiliations:** ^1^ Harold Simmons Comprehensive Cancer Center, UT Southwestern Medical Center, Dallas, TX, USA; ^2^ Hamon Center for Therapeutic Oncology Research, UT Southwestern Medical Center, Dallas, TX, USA; ^3^ Department of Immunology, UT Southwestern Medical Center, Dallas, TX, USA; ^4^ Department of Molecular and Cellular Medicine, Texas A&M University Health Science Center, College Station, TX, USA

**Keywords:** phosphatidylserine, extracellular vesicles, ovarian malignancies, exosomes

## Abstract

There are no suitable screening modalities for ovarian carcinomas (OC) and repeated imaging and CA-125 levels are often needed to triage equivocal ovarian masses. Definitive diagnosis of malignancy, however, can only be established by histologic confirmation. Thus, the ability to detect OC at early stages is low, and most cases are diagnosed as advanced disease. Since tumor cells expose phosphatidylserine (PS) on their plasma membrane, we predicted that tumors might secrete PS-positive exosomes into the bloodstream that could be a surrogate biomarker for cancer. To address this, we developed a highly stringent ELISA that detects picogram quantities of PS in patient plasma. Blinded plasma from 34 suspect ovarian cancer patients and 10 healthy subjects were analyzed for the presence of PS-expressing vesicles. The nonparametric Wilcoxon rank sum test showed the malignant group had significantly higher PS values than the benign group (median 0.237 *vs*. -0.027, *p*=0.0001) and the malignant and benign groups had significantly higher PS values than the healthy group (median 0.237 *vs* -0.158, *p*<0.0001 and -0.027 *vs* -0.158, *p*=0.0002, respectively). ROC analysis of the predictive accuracy of PS-expressing exosomes/vesicles in predicting malignant against normal, benign against normal and malignant against benign revealed AUCs of 1.0, 0.95 and 0.911, respectively. This study provides proof-of-concept data that supports the high diagnostic power of PS detection in the blood of women with suspect ovarian malignancies.

## INTRODUCTION

The detection of tumor-specific signatures in patient plasma has recently come to the forefront of cancer diagnosis. Data obtained from circulating tumor cells (CTC) [1], tumor-derived DNA (ctDNA) [2], and mRNA in tumor-educated platelets (TEP) [3] can be diagnostic for cancer and help pinpoint the location of tumors. Although specific tumor markers can be indicative of cancer type, a surrogate pan cancer-specific marker could be useful in the general diagnosis of cancer, differentiate between benign and malignant status of uncertain radiographic/sonographic lesions and serve as a predictive marker for recurrence and response to therapy. This is particularly relevant for ovarian cancers where there is currently no routine screening test. Moreover, once diagnosed, clinical staging is frequently ambiguous with many patients being diagnosed at advanced stages leading to poor survival rates.

Exosomes are 100-200 nm vesicles that are secreted to the extracellular space and peripheral circulation by most cells. They are formed by the inward budding of plasma membrane-derived multivesicular bodies that entrap nucleic acid and protein-rich cytosol. In addition to many exosome-specific proteins, these particles contain miRNA and a repertoire of protein signatures that are diagnostic for specific tumor types [4, 5]. Several studies have indicated that, in contrast to normal non-tumorigenic cells, tumor cells expose the phospholipid phosphatidylserine (PS) at the cell surface [6–10]. Because exosome membranes are derived from plasma membrane of the parent cell, these findings raise the possibility that unlike exosomes released from normal cells, exosomes derived from tumor cells, might expose PS. Indeed, PS-expressing exosomes are secreted from *in vitro* cultivated ovarian carcinoma (OC) cell lines and are found in ascites from OC patients [11–13]

Because PS on cell surfaces appears to be primarily a property of tumor cells and cells undergoing apoptosis, we determined if the presence of PS-expressing extracellular vesicles (EV) [14] in patient blood might be diagnostic for cancer. Towards this, we used an engineered, multivalent PS-specific antibody to develop a highly sensitive and quantitative ELISA for the detection of picogram amounts of PS in plasma. In a blind retrospective study carried out in accordance with guidelines for reporting of tumor marker studies (REMARK) [15] and standards for reporting diagnostic test accuracy (STARD) [16], we show that the presence of PS in blood accurately detects ovarian cancers and differentiates between patients with benign and malignant disease. These data suggest that the presence of PS in patient blood is diagnostic for cancer.

## RESULTS

### Expression of PS in tumor exosomes

To confirm that PS-expressing exosomes found in OC patient ascites are derived exclusively from tumor cells, exosomes from OC and mesothelial cell lines established from ascities obtained from the same patient were assessed for PS on the exosome surface by FACS and by PS-dependent acetate-mediated precipitation [17]. Figure 2 shows that, in contrast to OC exosomes, FITC-annexin 5 did not bind to exosomes from mesothelial cells (Figure 2A) nor were they precipitated with acetate (Figure 2B) suggesting that only tumor cell-derived exosomes expose PS. To confirm that the inability to precipitate and label normal cell-derived exosomes with annexin 5 was because they do not display PS, PS on tumor exosomes was hydrolyzed with phospholipase C and confirmed PS-free by flow cytometry (Figure 2C). The PS-negative (lipase-treated) population was then labeled with N-Rho-PE, (red fluorescence) and the PS-positive population with N-NBD-PE (green fluorescence) and precipitated with acetate [23]. Figure 2D shows high levels of fluorescence for the individual and mixed populations before acetate treatment (upper panels). After acetate precipitation, however, only the PS-positive, NBD-labeled exosomes were recovered from the resuspended pellet (lower panels). Taken together, these data confirm that, in contrast to normal cell-derived exosomes, only tumor cell-derived exosomes expose PS.

**Figure 1:**
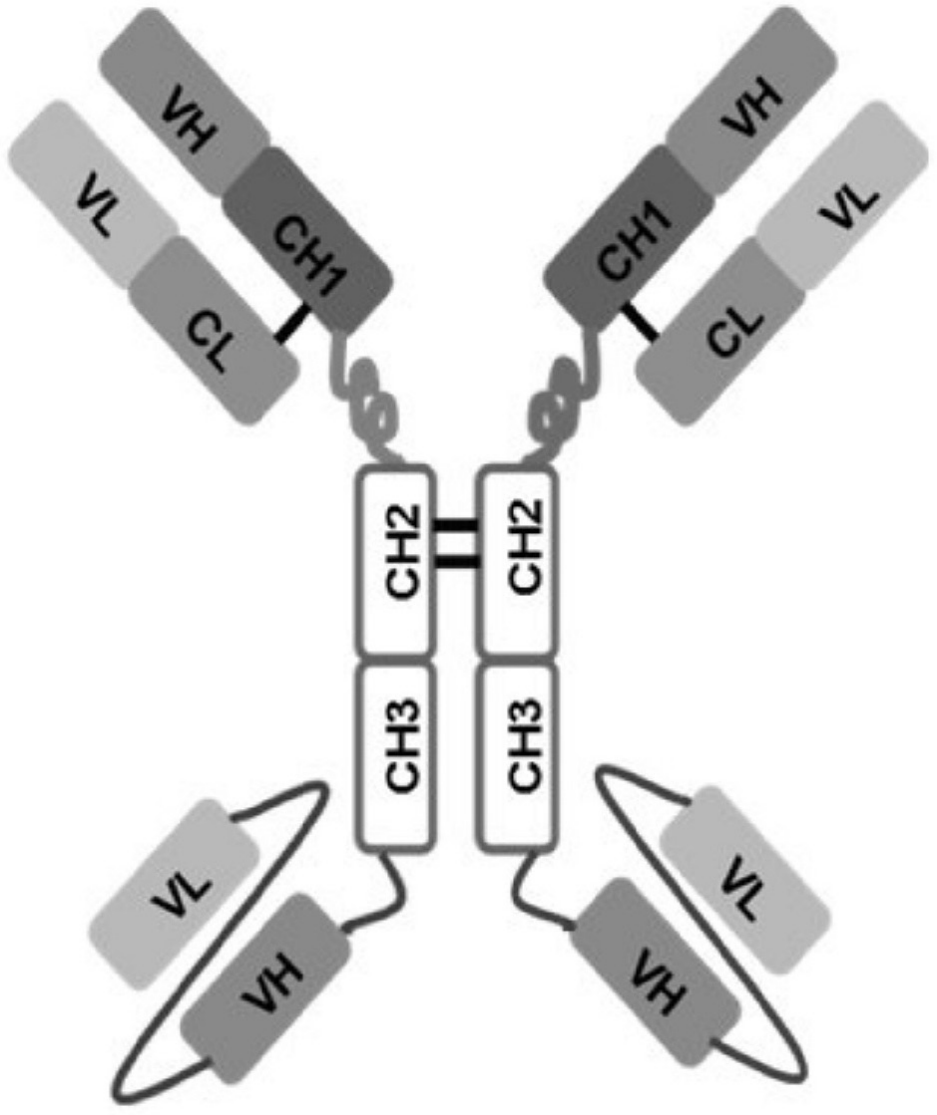
Schematic representation of 1N11-T comprising 1N11 scFv linked to CH3 domain of 1N11 by Gly-Ser-Ser linker

**Figure 2:**
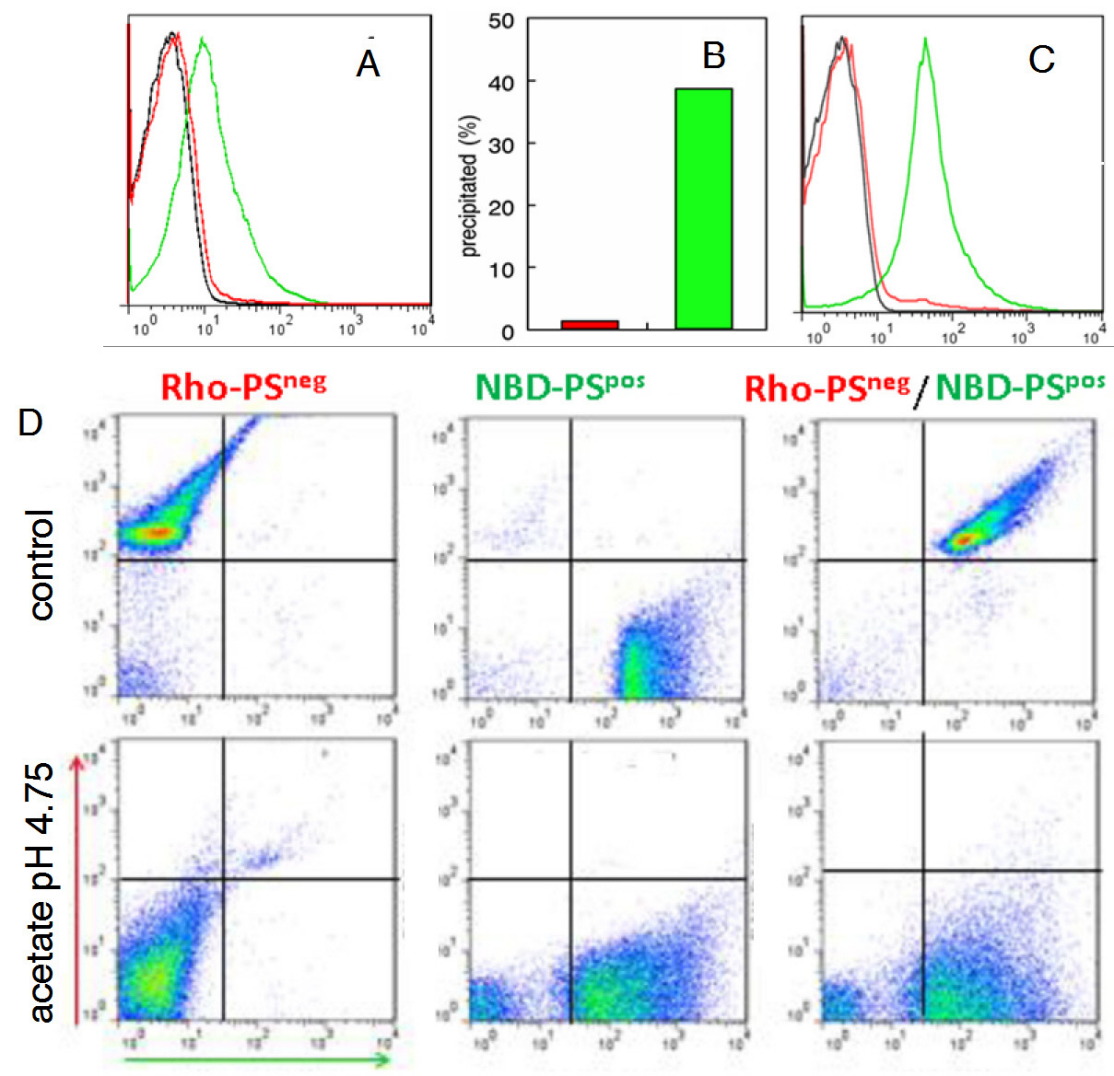
Tumor exosomes express PS **A**. Exosomes from cultured OC cells (green) and mesothelial cells (red) were collected by ultracentrifugation from cell supernatants. The pelleted exosomes were coupled to latex beads and incubated with FITC annexin 5 in Ca^2+^ containing buffer and analyzed by flow cytometry. Black, control BSA blocked beads; red, mesothelial cell exosomes; green, OC exosomes. **B**. Percent of exosomal protein recovered after acetate precipitation (0.1M, pH 4.75) of mesothelial cell-derived exosomes (red) and OC-derived exosomes (green). **C**. Flow cytometry analyses of PS-positive tumor exosomes (green) and PS-positive exosomes incubated with phospholipase C (red) to hydrolyze PS. Negative control (black). **D**. PS-positive and PS-negative exosomes from (C) were labeled with N-NBD-PE and N-Rho-PE, respectively. The indicated populations were then precipitated with Na acetate (0.1 M. pH 4.75), resuspended in buffer, coupled to latex beads and analyzed by flow cytometry

### PS-expressing exosomes in blood are a cancer biomarker

**Figure 3:**
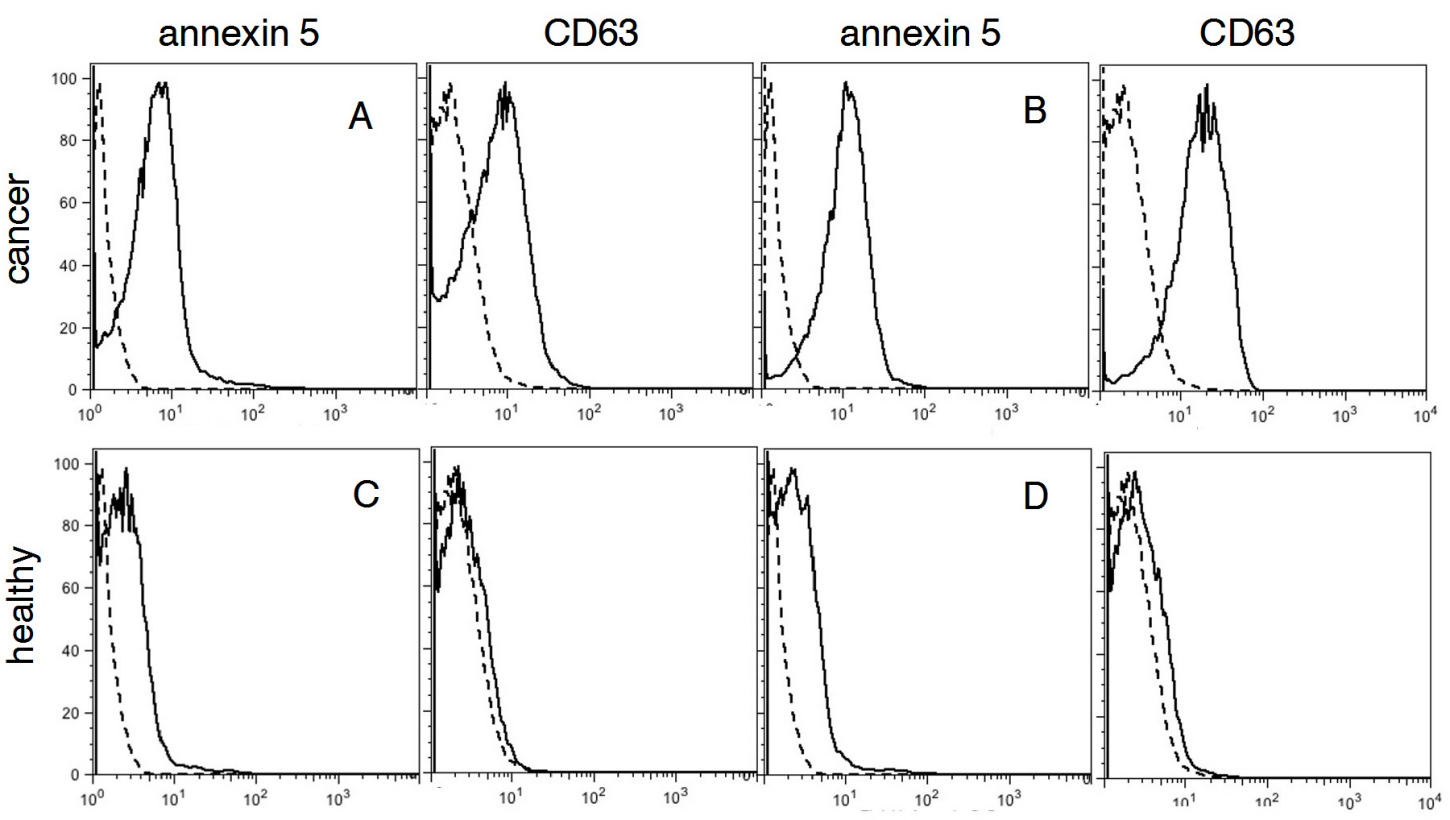
FACS analysis for PS and CD63 in plasma **A**. and **B**. confirmed cancer patients (red), **C**. and **D**., healthy individuals (green). Negative controls are shown in black.

To determine the nature of the PS-expressing extracellular vesicles captured with 1N11-T, plasma from healthy individuals and confirmed OC patients were incubated with 1N11-T-beads and analyzed for PS and CD63, a specific exosome marker [18]. The results shown in Figure 3 indicate that the PS-expressing EV captured with the 1N11-T beads from cancer patients were CD 63 positive (Figure 3A and 3B). These data indicate that the captured PS-expressing EV were most likely tumor-derived exsosomes. Importantly, PS-expressing EV's were not captured from plasma obtained from healthy individuals (Figure 3C and 3D).

**Figure 4:**
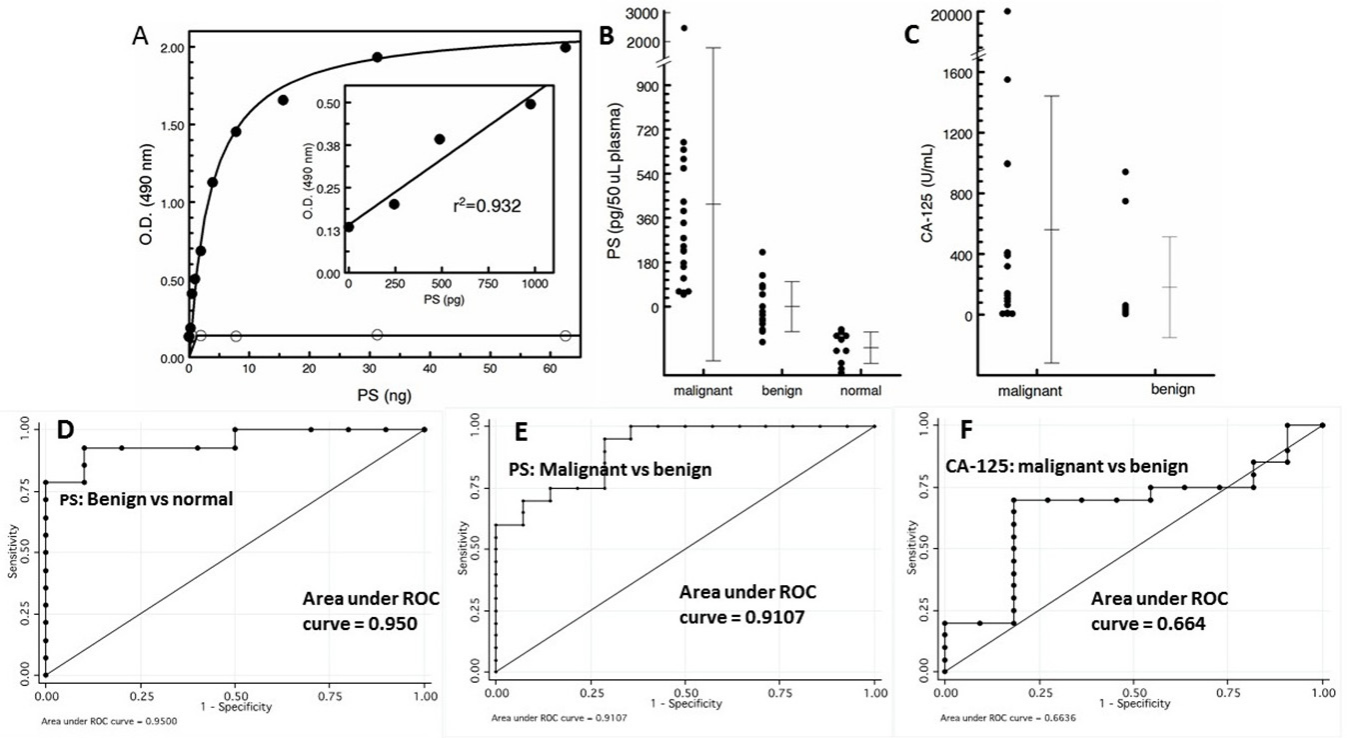
PS expressing exosomes in blood are a cancer biomarker **A**. Standard curve generated from the indicated amounts of PS expressed on the outer leaflet of LUV generated from PS/PC (wt/wt) (solid circles) and PC alone (open circles). For the 100% PC control vesicles, the total amount of added phospholipid was 4X the amounts indicated on the abscissa for PS. **B**. PS-expressing tumor exosomes in patient plasma. **C**. Patient CA-125 levels. ROC plots for PS for benign *vs* normal **D**., malignant *vs* benign **E**. and CA-125 plot for malignant *vs* benign **F**.

To quantify exosomal PS by ELISA using the tetravalent, PS-specific antibody 1N11-T (Figure 1), standard curves were generated from graded amounts of LUV containing 50% PS in PC (wt/wt). The ELISA data presented in Figure 4A shows that curves generated from vesicles containing PS reached saturation at > 10 ng PS. Importantly, excellent linearity was obtained in the range of 0 - 1000 pg PS. No binding was observed with LUV that did not contain PS (Figure 4A).

Analysis of plasma showed that the levels of PS-expressing exosomes distinguised between patients with histologically confirmed ovarian cancer (*n* = 20) and patients with benign masses (*n* = 14) and normal healthy individuals (*n* = 10) (Figure 4). Blood PS levels in patients with malignant disease was significantly higher (mean value of 415 pg/50 µL) than the levels of exosomal PS in the plasma of patients with benign disease (mean value of -1.0 pg/50 µL) which were higher than the levels found in normal, tumor-free individuals (mean value of -168 pg/50 µL). Interestingly, almost half the patients with benign disease had no detectable PS as did 100% of the individuals in the normal group (Figure 4B). The nonparametric Wilcoxon rank sum test showed the malignant group had a significantly higher marker value than the benign group (median 0.237 *vs*. -0.027, *p* = 0.0001) and both the malignant and benign groups had significantly higher marker values than the healthy tumor-free group (0.237 *vs* -0.158, *p* < 0.0001 and -0.27 *vs* -0.158, *p* = 0.00024, respectively). Receiver Operating Characteristic (ROC) analysis were performed to determine the accuracy, sensitivity, and specificity of PS in predicting malignant against healthy, benign against healthy and malignant against benign. ROC curves were also plotted and the optimal cutoff value for PS was determined. ROC analysis of predictive accuracy of malignant against normal revealed an area under the curve (AUC) of 1.0, with an optimal cutoff of -0.093 and corresponding sensitivity of 1.0 and specificity of 1.0 (not shown). ROC analysis of benign against healthy revealed an AUC of 0.950, with an optimal cutoff of -104, and corresponding sensitivity of 0.929 and specificity of 0.900 (Figure 4D), while ROC analysis of malignant against benign revealed an AUC of 0.911, with an optimal cutoff of 0.055 and corresponding sensitivity of 0.950 and specificity of 0.714 (Figure 4E). These results show that the predictive accuracy of PS was generally excellent. The nonparametric Wilcoxon rank sum test of CA-125 levels showed there was no significant difference between the malignant and the benign groups (median 118.95 *vs*. 43.5, *p* = 0.137) (Figure 4C) while the median value of the benign group (43.5) was consistent with published normal CA-125 values [19]. Indeed, for CA-125, ROC analysis of predictive accuracy revealed an AUC of only 0.664, with an optimal cutoff of 68.5, and corresponding sensitivity of 0.700 and specificity of 0.818 (Figure 4F).

**Figure 5:**
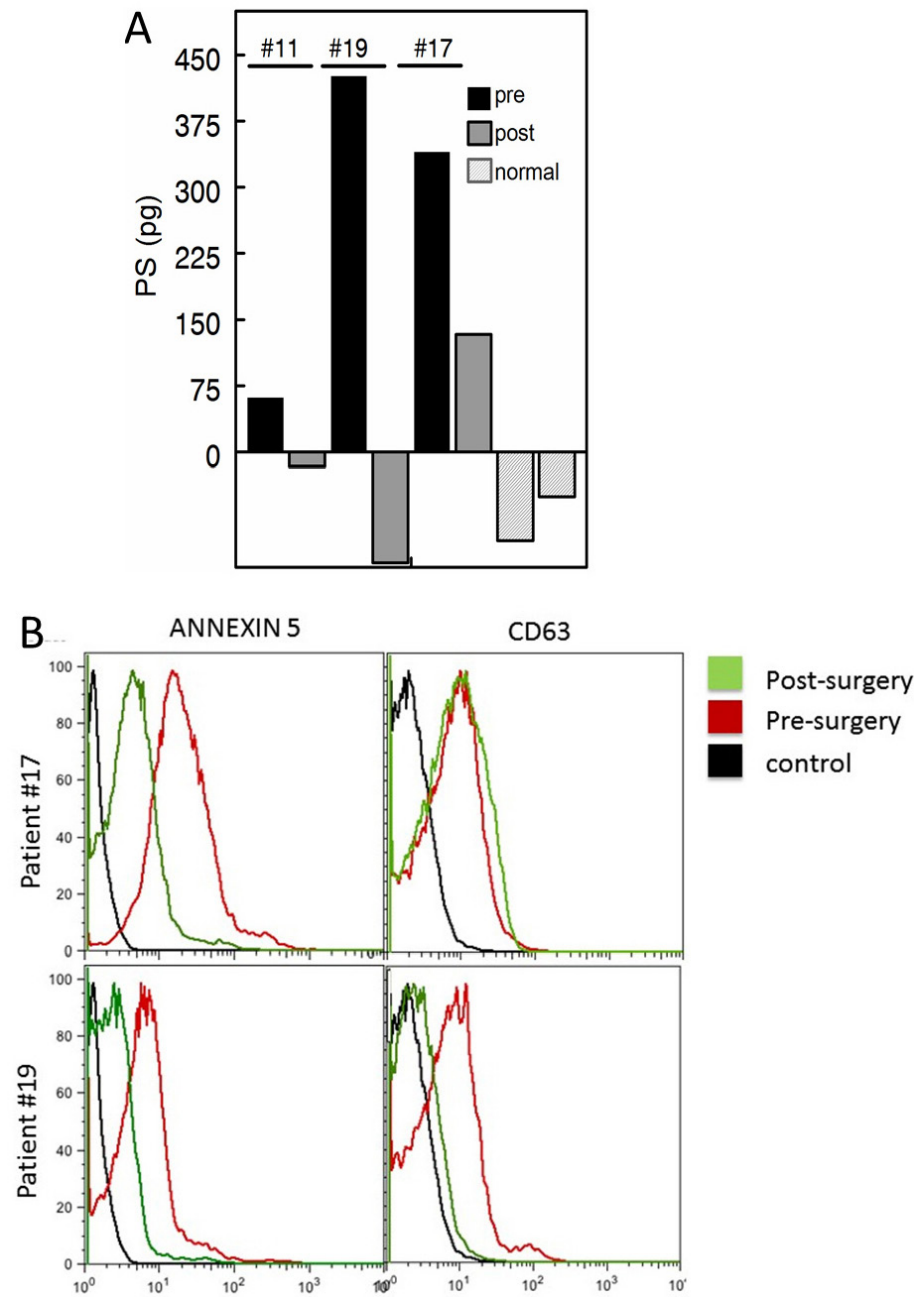
Pre and post surgery PS levels **A**. Blood PS exosome levels of patients pre surgery (black) and ~ 6 months post-surgery (dark grey). Representative normal controls are shown in light grey. Patients with the low post-surgery levels (patient ID's #11 and #19, Table 1) were confirmed no evidence of disease. The patient with the high post-surgery level (ID #17, Table 1) was clinically confirmed to have recurrent disease. **B**. FACS analysis for PS and CD63 levels of plasma exosomes from patients #'s 17 and 19. Post surgery (green). pre-surgery (red) and negative controls (black).

A blinded longitudinal study of blood collected from three patients ~6 months post surgery showed no detectable PS in the plasma of two patients. A third patient, however, still showed significantly elevated amounts of PS (~133 pg *vs* a pretreatment value of 340 pg) suggestive of recurrance or residual disease (Figure 5A). Clinical follow-up confirmed the analysis; the first two patients had no evidence of disease whilst the third patient did recur. Interestingly, post-surgical CA125 levels of patients #11 and #19 were within “normal” range (11.5 and 17.0), while patient #17 was borderline positive (40.6). FACS analysis confirmed that the PS-free patient (patient #19) had no residual double positive (PS + CD63) exosomes in her plasma while the patient with the relatively high residual PS (patient # 17) also expressed significant amounts of CD63-positive (tumor) exosomes (Figure 5B).

## DISCUSSION

While established screening programs for breast, cervical and colorectal cancer can detect asymptomatic disease, most women with ovarian cancers remain asymptomatic at potentially curable stages coming to clinical attention only after symptom emergence. Although a minority of these cases can be investigated with blood CA-125 levels and transvaginal ultrasound, neither has the sensitivity or specificity for detecting early stage asymptomatic disease [20–22]. Unfortunately, ~50% of stage I ovarian cancers have normal CA-125 levels.

Additionally, the increasing use of highly sensitive whole-body MRI and MRA imaging technologies has resulted in an epidemic of “incidentalomas” - radiographic findings of unclear clinical significance, with detection of unexpected findings in 68% of otherwise healthy adults that in many cases leads to further imaging and surveillance [23]. Clearly, a highly sensitive, accurate and reproducible biomarker could eliminate many of these issues by shortening the time to diagnosis, resulting in earlier treatment and significantly better outcomes.

There is increasing evidence that tumor cells expose PS on their surface [9, 13] by a mechanism that is unrelated to PS externalization commonly seen in dying apoptotic cells [9]. Physiologically, cell surface PS plays critical roles in the recognition and removal of effete and dying cells from the host and in quelling immune responses against “self” [24]. Because many constituents of exosomal membranes are derived from the plasma membrane of the originating parent cell, we hypothesized that “liquid biopsies” of cancer patient blood, but not blood from tumor-free individuals, would contain PS-expressing tumor-derived exosomes. Although a myriad of potential cancer- and tumor-type-specific exosome biomarkers in blood have been identified [4], there are no studies showing that PS expression on the surface of blood exosomes is a reliable surrogate for the detection of malignancies. While there are studies showing the presence of large plasma membrane-derived PS-expressing EV from apoptotic cells [25], red cells [26], platelets [27] and immune cells [28] in patient blood [29], only one report shows a positive relationship between the concentration of PS-positive EV and tumor burden [30].

Previous studies have established that cancer patients have significantly higher concentrations of plasma exosomes than normal, tumor-free individuals [31–34]. Our data indicating that only cancer patients have highly elevated levels of PS-expressing exosomes in their plasma suggest that the source of PS is derived exclusively from cancer cells. Since we were unable to detect PS-positive exosmes in the healthy cohort, if there are blood exosomes from non-tumorigenic cells that display PS, their concentrations are likely far below the picogram amounts we found in patient blood. To ensure our analysis was based on the detection of PS from tumor cell-derived exosomes, large EV were removed from the blood by 12,000g centrifugation prior to analysis. Additionally, the presence of exosomes in the plasma supernatants was confirmed by the presence of the exosome-specific marker, CD63 (Figures 3 and 5B). Based on findings that OC patient ascites contain tumor exosomes that display PS on their surface [11–13], we used ovarian cancers as a model for the development a very high stringency ELISA that both selectively binds and detects only PS-expressing exosomes.

The data summarized in Figure 4 show that quantification of PS-exosomes in blood distinguishes, with 100% accuracy, healthy tumor-free individuals from patients with ovarian malignancies (AUC = 1.0). Although there is some overlap in results obtained from healthy and patients with benign lesions (AUC = 0.950) and patients with benign lesions *vs* malignancies (AUC = 0.911), analysis of all patients’ *vs* healthy resulted in AUC of 0.979 (not shown). It should be noted, however, that while the relative differences in marker values obtained between the malignant, benign and healthy cohorts were consistently reproducible and highly significant, the amounts of PS quantified on the exosome surfaces may not reflect the actual amounts of PS. There are several reasons for this: 1) The transbilayer distribution of PS assumed to be 50%/leaflet of the total PS in the standard LUV may be an over- or underestimate and, 2) The planar distribution of PS in the LUV might not reflect the distribution of PS in the exosome membrane. In principle, this could affect the efficiency of capture to the plates and detection with annexin A5. These potential differences could explain the negative values of PS obtained in the normal cohort. Nonetheless, these data show that the PS exosome assay accurately overcomes much of the uncertainty of distinguishing healthy women from women harboring ovarian malignancies irrespective of tumor type (Table 1).

In summary, this study provides proof-of-concept data that supports the high diagnostic power of PS-expressing tumor exosome detection in blood from women with suspect ovarian malignancies. Ultimately, these studies could lead to earlier stage diagnosis, substantial cost savings, reduced patient exposure to radiation and invasive procedures, and improved clinical outcomes. The assay might also find utility in patients with radiographic abnormalities, even before clinical detection. Indeed, an accurate biomarker predicting the likelihood of malignancy would be extremely beneficial to such a population since they often face long periods of anxiety and uncertainty inherent to a “wait and watch” approach. Finally, if PS-exosome diagnostics are confirmed in a large study to be an accurate and reproducible biomarker of ovarian malignancies, the assay could be applied to the early detection of other visceral malignancies.

**Table 1:** Characteristics of study cohorts

MALIGNANT										
Patient No.	Age	Race	Histology	Stage	Grade	Other cancers	Comorbidities	CA-125	Exosome PS (ρg)	Other abnormal tumor markers
1	55	White	High grade Serous	IIIC	3	-	HTN	404	637	-
2	48	Black	High grade Serous	IIIB	3	-	HTN,GERD,Glaucoma	7	161	CA 19-9: 31 CEA: .8
3	62	Black	Granulosa Cell Tumor	IA	NA	-	HTN	7.6	667	INHB: 4460
4	44	Hispanic	Serous Borderline tumor	IIA	NA	-	Hyperthyroidism, Graves’ disease, HTN, osteoporosis	997	388	-
5	48	Hispanic	Endometrioid Adenocarcinoma	IIIC	3	-	GERD, hyperthyroidism, diabetes	12.4	561	-
6	47	Hispanic	High grade Serous	IV	3	-		1550	56	
7	54	White	Clear Cell carcinoma	IC	3	-	Diabetes, HTN, ESRD	993	599	CA 19-9: 696 CEA: 17.2
8	38	Asian	High grade Serous	IIB	3	-	Eczema	65.1	244	
9	59	White	High grade Serous	IIIB	3	-	Renal transplant, immunosuppression	128	176	-
10	59	Hispanic	High grade Serous	-	3	-	Diabetes	>20000	61	-
11	52	Black	Carcinoma of ovary	IIIC	3	-	HTN	411.1	61	-
12	47	Black	Granulosa Cell Tumor	IA	NA	-	-	89.8	279	
13	52	Black	Granulosa Cell Tumor	IC1	NA	-	CHF, HTN, GERD, Diabetes	7	2971	INHB: 1380
14	37	Hispanic	Mucinous Adenocarcinoma	IC2	2	-	-	106.6	53	CA 19-9: 454 CEA: 40.4
15	58	Asian	Endometrioid Adenocarcinoma	IA	1	-	GERD, asthma	141.9	229	CA 19-9: 414
16	52	Hispanic	High grade carcinoma	IIIC	3	-	-	11.7	49	
17	55	White	High grade Serous	IIIC	3	-	-	3456	340	-
18	47	Hispanic	High grade Serous	IIIC	3	-	-	390.3	225	-
19	52	Black	High grade Serous	IIIC	3	-	HTN	109.9	426	
20	43	Hispanic	Mucinous Borderline	IC	NA	-	-	319.2	114	CA 19-9: 6253
**BENIGN**										
**Patient No**.	**Age**	**Race**	**Histology**	**Stage**	**Grade**	**Other cancers**	**Comorbidities**	**CA-125**	**Exosome PS (ρg)**	**Other abnormal tumor markers**
21	39	White	NED	-	-	Breast	Prior chemotherapy	-	126	
22	21	Hispanic	Mucinous Cystadenoma	-	-	-		62	49	
23	28	Black	Benign Mucinous Cystadenoma	-	-	-	-	43.5	85	CEA: 67.5
24	70	Hispanic	Benign Serous Cystadenoma	-	-	-	Osteoporosis	52.5	-101	
25	35	Hispanic	Leiomyomas	-	-	-	-	749	-144	-
26	43	White	Benign : Endometrioisis	-	-	-	GERD	54	-50	
27	39	Hispanic	Benign : Endometrioisis	-	-	-	-	942	0	CA 19-9: 287-
28	42	White	Benign Cyst	-	-	Melanoma	-	16.9	-33	
29	41	Hispanic	NED	-	-	Breast	-	<5.5	-70	-
30	64	Black	Benign Cystadenofibroma	-	-	-	Diabetes, HTN	24.4	-20	
31	46	Hispanic	Benign Cyst	-	-	Breast	-	-	77	
32	54	Black	Benign Cyst	-	-	-	HTN, Hyperlipidemia, arthritis	33.7	-94	
33	42	Asian	NED	-	-	-	-	8.6	-57	-
34	52	Black	Benign	-	-	-	HTN, sarcoidosis, asthma	-	221	-

## MATERIALS AND METHODS

### Patient samples

Blood was collected from patients just prior to scheduled exploratory surgery for suspect ovarian malignancies and from healthy sex matched donors obtained from the UT Southwestern Gynecologic Oncology clinics and UT Southwestern Biomarker Research Core, respectively. All samples were collected in accordance with UT Southwestern Institutional Review Board (STU 062010-201 and STU 092014-013). All individuals were selected at random without any inclusion or exclusion criteria. Patients’ informed consent was obtained before blood collection. Blood was collected in K_3_EDTA vacutainers. Platelet poor plasma was prepared by centrifugation for 10 min at 700g to remove blood cells. Plasma was collected and again centrifuged at 12,000g for 5 min to remove platelets and large extracellular vesicles. The plasma was stored at -20°C. All samples were coded and analyzed blinded. Samples were unblinded and the experimental data and corresponding clinical parameters and pathologic diagnosis of each patient was revealed at the end of all the assays. Patients with confirmed malignancies irrespective of tumor type were grouped as “malignant” and patients with benign tumors or “no evidence of disease” (NED) were grouped as “benign” (Table 1). Patients with the following diagnosis were analyzed (Table 1): high grade serous (*n* = 9), granulosa cell tumor (*n* = 3), serous borderline (*n* = 1), endometrioid adenocarcinoma (*n* = 2), clear cell carcinoma (*n* = 1), carcinosarcoma (*n* = 1), mucinous adenocarcinoma (n = 1), high grade carcinoma (*n* = 1), and mucinous borderline (*n* = 1).

### Expression of an engineered tetravalent antibody for PS-detection

Monoclonal 1N11 is a human IgG1λ that binds PS through the PS-specific plasma protein β2GP1 [35]. A tetravalent variant of 1N11 (1N11-T), with four binding sites per molecule was designed to generate a high avidity PS binding agent (Figure 1). To generate a 1N11-T heavy chain expression construct, a design similar to that described for tetravalent bispecifics was used [36, 37]. A linker sequence encoding (Gly-Ser-Ser) and containing a unique XhoI site was inserted at the 3′ end of the heavy chain gene of 1N11 using a designed oligonucleotide and the PCR. The gene encoding the 1N11 single chain (sc)Fv flanked by XhoI sites and with codons encoding a (Gly_4_Ser)_3_ linker peptide between the V_H_ and V_L_ domain genes was ordered from Genescript (New Jersey). An expression construct for the full length 1N11 heavy chain (human IgG1) linked to the 1N11 scFv, using pOptiVEC TOPO (Invitrogen) as vector, was generated using standard methods. The expression construct for the 1N11 light chain was generated by linking the gene encoding the 1N11 light chain variable domain, using splicing by overlap extension [38], to the human Cλ gene using pcDNA3.3-TOPO (Invitrogen) as vector. Reverse transcriptase PCR was used to isolate the Cλ gene from RPMI 8226 cells (purchased from the ATCC). The 1N11 heavy chain and scFv fragments were digested by XhoI and purified. The fragments were ligated and transformed into oneshot TOPO competent E. *coli*. Expression plasmids for 1N11-T in stably transfected CHO cells were generated: The light chain expression construct was transfected into CD/DG44 CHO cells (Life Technologies) using electroporation and selected with CD/DG44 CHO medium containing 500 μg/ml geneticin without HT supplement. The heavy chain expression construct was then transfected into a light chain expressing CD/DG44 CHO clone that showed the highest level of expression. Heavy chain transfectants were selected with Opti-CHO Medium containing 500 μg/ml geneticin. Supernatants of clones were screened by sandwich ELISA using goat human Fab-specific antibody as capture antibody and goat human Fc-specific antibody conjugated to horseradish peroxidase as detection antibody. The clone expressing the highest levels of 1N11-T was expanded and cultured in increasing concentrations of methotrexate, to induce gene amplification. Clones were expanded in shake flasks and antibody was purified with protein G-Sepharose.

### Isolation of ovarian carcinoma cells and normal mesothelial cells

Ovarian tumor and mesothelial cell cultures [39] established from the same patient's ascites were kindly provided by Dr. Adi Gazdar (UT Southwestern Medical Center). Briefly, ascites was centrifuged, the cell pellet was resuspended in medium and the tumor clusters were allowed to sediment while the mesothelial cells remained in suspension. After several cycles of differential sedimentation, differential plating was used to further separate the two populations. Each cell type was then cryo-preserved and grown in ACL4 medium.

### Cell lines

Cells (~25 x10^6^ in 15 mL media) were seeded into the lower chamber of CELLine AD 1000 flasks (Integra Biosciences AG) that contained 250 mL media in the upper chamber [40]. Conditioned medium (~15 mL) containing the secreted exosomes were collected from the lower chamber weekly. Typical yields were 75 - 125 µg of exosomes/mL medium.

### Exosome isolation from cell lines

Conditioned medium was cleared of cells, cell debris and large extracellular vesicles by sequential centrifugation at 700 g for 30 min followed by 12,000 g for an additional 30 min. Exosomes were collected from the cleared supernatants after centrifugation at 70,000 g for 2 hrs and one wash in HEPES-saline (NaCl 150 mM, HEPES, 20 mM, EGTA 2 mM, pH 7.6). The pellets were resuspended in ~0.5 mL HEPES-saline. Exosome quantity was estimated by BCA assay.

### Hydrolysis of exosomal phospholipids and fluorescent labeling of exosomes

Tumor exosome surface phospholipids (50 µg protein) were hydrolyzed with *Bacillus cereus* phospholipase C (50 U, Calbiochem) in 1.0 mL of Tris buffer (0.1 M) containing 0.02 M CaCl_2_ at 20°C overnight. The lipase was removed by washing at 70,000 g for 2 hrs.

Purified PS-expressing and PS-free (phospholipase-treated) tumor exosomes were labeled with N-NBD-phosphatidylethanolamine (N-NBD-PE) and N-rhodamine-phosphatidylethanolmine (N-Rho-PE), respectively. Briefly, 1 µg of each probe in 50 µL ethanol was deposited on the bottom of a glass tube and 25 µg of exosomes (in 1.0 mL HEPES-saline) were injected into the tube under vigorous vortexing. The exosomes were then precipitated with acetate (0.1 M pH 4.75) [17]. The precipitated vesicles were collected by centrifugation (1,000 g), resuspended in PBS, coupled to latex beads and analyzed by flow cytometry.

### Flow cytometry

#### Exosomes from cell lines

Tumor exosomes (10 µg) in 0.5 mL HEPES-saline were mixed overnight at 4°C with 5 µL of 4 µm aldehyde-activated latex beads (4% w/v) (Invitrogen). The beads were blocked with 1% BSA for 1 hr. After washing (5,000 g for 5 min.) the beads were resuspended in HEPES-saline containing Ca^2+^ (1 mM) and FITC-labeled annexin 5 (BD Biosciences). Samples were screened with a BD Biosciences FACS Calibur. Data was analyzed using FlowJo.

#### Exosomes from patient samples

5 µL of 4 µm aldehyde-activated latex beads (4% w/v) (Invitrogen) were incubated with 1N11-T (12.5 µg) at 4°C overnight. The beads were then washed, blocked for 1 hr with 1% BSA and mixed with 300 µL of a 1/6 dilution of patient plasma for 2 hrs at 20°C. The beads were then washed with HEPES-saline and a 1/100 dilution of mouse anti-human CD63 antibodies (Sigma #SAB4700215) for 30 min. The beads were then washed and stained for mouse Ig and PS with a 1/500 dilution of FITC-goat anti-mouse Ig (Jackson Labs #115-095-166) and a 1/100 dilution of Cy5-annexin 5 (Biovision #1013-200) in HEPES-saline containing Ca^2+^ (1 mM). Samples were screened and analyzed as described above.

### PS liposomes

Large unilamellar vesicles (LUV) were prepared by extrusion through 0.1 µm membranes (Avanti mini-extruder, Avanti Polar Lipids, Birmingham, AL). Briefly, liposomes were prepared by mixing PS with phosphatidylcholine (PC) (0.5 mg each) in CHCl_3_. The lipids were dried under N_2_, resuspended in 1 mL of HEPES-saline and extruded though the membrane 15 times. For standard curves, we assumed that half the PS localized in the outer leaflet of the LUV. Thus, 1 mg of LUV containing 50% PS would present with 250 µg of PS/mL accessible for binding.

### ELISA assay

Immunolon 1B U-bottomed ELISA plates were coated with 100 µL of 1Ν11−Τ (10 µg/mL) overnight at 4°C. The plates were then washed with PBS and blocked with BSA (2% in PBS) at 37°C for 1 hr. The plates were again washed and the wells were loaded with 100 µL of PS/PC (1/1) LUV (double diluted from 1000 ng PS/mL) or 100 µL of a 1/2 dilution of plasma in PBS. The plates were then incubated at 37°C for 3 hours and washed with PBS. 100 µL of a 1/1000 dilution of biotinylated annexin 5 (Life Technologies #A13204) in HSCa buffer (10 mM HEPES, 140 mM NaCl, 2.5 mM CaCl_2_ and 2% BSA) was added for 1 hr at 20°C. The plates were then washed with the same buffer and HRP-streptavidin (1/1000 100 µL in HSCa) was incubated for 10 min at 20°C. After washing with HSCa, the plates were developed with 100 µL of OPD (0.5 mg/mL) and H_2_O_2_ (1 µL/mL) in 50 mM citrate phosphate buffer, pH 4.3. The reaction was stopped with 0.18 M H_2_SO_4_ and absorbance at 490 nm was determined in a plate reader.

### Statistical analysis

The nonparametric Wilcoxon rank sum test was used to evaluate differences in PS values or CA-125 values between the malignant and benign groups, between the malignant and normal tumor-free groups, and between the benign and normal tumor-free groups. Receiver Operating Characteristic (ROC) curves were constructed, and the area under the curve (AUC) was calculated to evaluate the accuracy, sensitivity, and specificity of PS or CA-125 in predicting malignant against benign tumors. The optimal cutoff point of PS or CA-125 was determined based on the Youden Index, and is defined as the biomarker value that maximizes the summation of (sensitivity+specificity-1). The sample size justification is not necessary due to the exploratory nature of this biomarker study. All statistical tests were two-sided, and a *P* < 0.05 was considered statistically significant. All analyses were performed using STATA (Release 14, College Station, TX).

## Abbreviations

AUC: area under the curve
EV: extracellular vesicles
LUV: large unilamellar vesicles
NBD-PE: NBD-labeled phosphatidylethanolamine
N-Rho-PE: rhodamine-labeled phosphatidyl-ethanolamine
OC: ovarian carcinoma
PS: phosphatidylserine
ROC: receiver operating characteristics
